# Prevalence of different variations of non-consented care during the childbirth process in Mexico by geographical regions: comparing ENDIREH survey data from 2016 to 2021

**DOI:** 10.1186/s12884-024-06549-1

**Published:** 2024-05-13

**Authors:** Marian Marian, Kathryn M. Barker, Elizabeth Reed, Amanda C. McClain, Rebecka Lundgren, Samantha Hurst, Ramona L. Pérez

**Affiliations:** 1 Herbert Wertheim School of Public Health and Human Longevity Science, University of California San Diego, 9500 Gilman Drive, La Jolla, San Diego, CA 92093 USA; 2 San Diego State University School of Public Health, San Diego, CA USA; 3 Center on Gender Equity and Health, University of California San Diego, La Jolla, San Diego, CA USA; 4 School of Exercise and Nutritional Sciences, San Diego State University, San Diego, CA USA; 5 Department of Anthropology, San Diego State University, San Diego, CA USA

**Keywords:** Obstetric violence, Non-consented care, Mexico, Delivery, Childbirth, Violence against women

## Abstract

**Introduction:**

Non-consented care, a form of obstetric violence involving the lack of informed consent for procedures, is a common but little-understood phenomenon in the global public health arena. The aim of this secondary analysis was to measure the prevalence and assess change over time of non-consented care during childbirth in Mexico in 2016 and 2021, as well as to examine the association of sociodemographic, pregnancy-, and childbirth-factors with this type of violence.

**Methods:**

We measured the prevalence of non-consented care and three of its variations, forced sterilization or contraception, forced cesarean section, and forced consent on paperwork, during childbirth in Mexico for 2016 (*N* = 24,036) and 2021 (*N* = 19,322) using data from Mexico’s cross-sectional National Survey on the Dynamics of Household Relationships (ENDIREH). Weighted data were stratified by geographical regions. We performed adjusted logistic regression analyses to explore associations.

**Results:**

The national prevalence of non-consented care and one of its variations, pressure to get a contraceptive method, increased from 2016 to 2021. A decrease in the prevalence was observed for forced contraception or sterilization without knowledge, forcing women to sign paperwork, and non-consented cesarean sections nationally and in most regions. Women between the ages of 26 and 35 years, married, cohabiting with partner, living in urban settings, who do not identify as Indigenous, and who received prenatal services or gave birth at the Mexican Institute of Social Security (IMSS) facilities experienced a higher prevalence of non-consented care. Being 26 years of age and older, living in a rural setting, experiencing stillbirths in the last five years, having a vaginal delivery, receiving prenatal services at IMSS, or delivering at a private facility were significantly associated with higher odds of reporting non-consented care.

**Conclusion:**

While a decrease in most of the variations of non-consented care was found, the overall prevalence of non-consented care and, in one of its variations, pressure to get contraceptives, increased at a national and regional level. Our findings suggest the need to enforce current laws and strengthen health systems, paying special attention to the geographical regions and populations that have experienced higher reported cases of this structural problem.

## Introduction

Obstetric violence, also known as disrespect and abuse during childbirth, is increasingly recognized as a global public health concern thanks to the growing body of research and documentation of women’s experiences with childbirth [1, 2]. Obstetric violence, a multifactorial phenomenon which can be both structural and interpersonal, involves any type of loss of autonomy, physical harm, or suffering during the prenatal, childbirth, and postnatal periods [3, 4]. Short- and long-term physical health outcomes have been found in women who have suffered from obstetric violence. Some of these include incontinence, breastfeeding problems, and complications during and after the delivery, such as episiotomies, postpartum hemorrhage, and obstructed delivery [5–7]. Mental health outcomes that have been associated with obstetric violence include postpartum depression, post-traumatic stress disorder, anxiety, guilt, and sadness [5, 6, 8]. Research has also shown that distrust and dissatisfaction with the health care system are exacerbated by experiences of obstetric violence [5, 9]. This in turn can lead to a delay or reduction in the use of healthcare services, which can negatively affect both the mother and the newborn child [5, 6]. 

The World Health Organization (WHO) advocates for the division of obstetric violence into seven distinct categories: physical care (such as beating and slapping), non-consented care (lack of informed consent for procedures), non-confidential care (lack of physical privacy and confidentiality of sensitive information), non-dignified care (e.g., intentional humiliation such as scolding and shouting at women), discrimination (commonly based on a woman’s ethnicity, race, economic status, educational level, religion, or age), abandonment of care (leaving a woman alone during labor and/or after delivery), and detention in facilities (e.g., confining the woman or infant at the clinic until the bill is paid) [10, 11]. Of these, non-consented care is particularly common in low- and middle-income countries, as women in these settings are often not informed about the risks and reasons for interventions during childbirth and are not asked for consent about the procedures to be completed during delivery [12]. Specifically, non-consented care during the childbirth process involves the absence of informed consent or of an information process for the pregnant person [10]. Different variations of the category of non-consented care during delivery include the administration of unconsented interventions such as forced cesarean sections (C-sections), forced contraceptive methods or sterilization, forced hysterectomies, and forced episiotomies during the absence of consent or even after refusal of the procedure, as well as forcing women to sign paperwork, to name a few [10, 13, 14]. Receiving information and having a supported informed consent process are critical components of a birth experience that is safe and offers quality care [12]. 

Obstetric violence is common in high-, middle-, and lower-income countries, such as Pakistan, Ethiopia, South Africa, and women from ethnic minorities in the United States, to name a few [15–18]. Notably, obstetric violence is a prevalent phenomenon in Latin America [13, 19], with an estimated 43% of women having experienced abuse and mistreatment during childbirth [19], and documented presence of non-consented care in several countries of this region [10]. Specifically, in Mexico, the prevalence of obstetric violence in the past fifteen years ranges from 6 to 33%, based on previous studies conducted at the city level in two cities in Central Mexico [20]. The denial of care to Indigenous women and unnecessary C-sections in this country are forms of obstetric violence particularly identified in the literature [11, 21–23]. Specific to non-consented care, a mixed-methods study completed in four hospitals across the Mexican states of Puebla and Chiapas found that more than 50% of women experienced non-consented care, as they did not receive adequate information for three invasive procedures (genital cleansing, genital shaving, and enema administration) and did not provide consent for them [20]. Episiotomies, manual uterine cavity revisions, and vaginal examinations are other procedures that have been found to be practiced in Mexico without the consent of female patients during childbirth [24, 25]. While the research on obstetric violence in this country has been documented in the past fifteen years, it has been mostly qualitative; however, quantitative studies have been completed at a local level in Mexico City and the states of Puebla, Chiapas, and Morelos [20, 26, 27]. Information on sociodemographic, pregnancy, and childbirth factors of women who have experienced non-consented care during childbirth in Mexico is restricted to the few local studies that have reported these characteristics [20, 28]. Due to the limited literature on non-consented care during childbirth, a gap exists in understanding the populations affected by this form of obstetric violence. Using national data is critical to capturing the magnitude and distribution of this form of violence at a country, state, and local level, as well as to better estimate the characteristics of the population affected by it and translate the findings into informed interventions and laws to address obstetric violence.

Awareness of obstetric violence has increased in Mexico in the past ten years [29, 30]. At the national level, in 2014, obstetric violence was categorized as a punishable offense [31]. In 2016, at the national level, changes were made to the law to improve the quality of care for pregnant women by emphasizing the inclusion of women in the decision-making process, eliminating any form of obstetric violence practices, and modifying the definition of pregnancy [32]. At the state level, in 2013, only four out of the 32 states in Mexico had a definition for obstetric violence in their laws [33]. As of 2021, 28 states Mexico had a definition of obstetric violence in their respective laws about access to life without violence [33]. This has resulted in norms and recommendations on best practices in delivery being followed more closely by health professionals [29]. Still, whether the prevalence of non-consented care has changed in Mexico is unknown.

While no standardized or validated tool to measure obstetric violence exists [34], Mexico has measured obstetric violence twice, in 2016 and 2021 [35, 36], through the National Survey on the Dynamics of Household Relations (Encuesta Nacional sobre la Dinámica de las Relaciones en los Hogares, ENDIREH, its Spanish acronym), a probabilistic household survey that uses an advisory committee of experts in violence against women that included academic, civil society, and governmental organizations in the creation of this instrument [13, 37]. This study sought to examine the prevalence of non-consented care, by type of non-consented care and by geographical region, among Mexican women for the years 2016 and 2021 using data from ENDIREH, which is representative at the national and state levels. We also aimed to determine if there is a difference in the prevalence of this specific form of obstetric violence between 2016 and 2021 and examine the association between sociodemographic, pregnancy, and childbirth factors with non-consented care. This will help identify any institutional, sociodemographic, or individual factors that could be associated with non-consented care. Our results will provide insight into the prevalence of non-consented care during childbirth in Mexico and help determine the geographical location and key socio-demographic characteristics of the women at greater risk of a specific form of obstetric violence.

### The term obstetric violence

The literature uses different names for the violence and abuse directed at women during childbirth. Obstetric violence, mistreatment during childbirth, and disrespect and abuse are the most common ones [38]. While mistreatment during childbirth is the term commonly used by the WHO [12], obstetric violence is the term generally used in Latin America [38]. Particularly in Mexico, this concept has been used by researchers since 1998 [13]. Because of these reasons and following the vocabulary used in the ENDIREH surveys to collect information on violence towards women during childbirth, the term used in this analysis was obstetric violence.

## Methods

### Study design and data source

This present study was a secondary cross-sectional analysis of the ENDIREH 2016 and 2021 data on one of the different types of violence against women this survey collects, obstetric violence. We used weighted regression analysis to examine the prevalence of a specific form of obstetric violence, non-consented care, among ENDIREH 2016 and 2021 respondents. The association of non-consent care with sociodemographic characteristics and pregnancy- and childbirth-related factors was also observed.

Since 2003, Mexico’s National Institute of Statistics and Geography (INEGI, its Spanish acronym), an autonomous public entity in charge of national surveys and census [39], has conducted ENDIREH to measure different forms of violence against women in the country, such as economic, sexual, psychological, physical, and patrimonial, and within different scopes of occurrence (community, family, partner, school, and at work) [13, 37]. The objective of ENDIREH is to estimate the prevalence of the different types of violence against women included in this survey, with the goal of eradicating these types of violence through the creation and implementation of informed public policies [36]. ENDIREH is a national and state-level representative cross-sectional survey conducted every four years by the Mexican government [13, 37]. Geographically, ENDIREH covers the population in the national, state, and national urban and rural regions [37]. The unit of observation is a private household and women ages 15 and older who live in that household [37]. Trained female data collectors surveyed, in person, only one woman per household among the households selected [40]. Three dimensions of obstetric violence —non-consented care, abandonment of care, and undignified care —were measured at a national representative level for the first time in Mexico through ENDIREH between October and November 2016 [13]. Between October and November 2021, ENDIREH measured obstetric violence for the second time in Mexico [36]. 

### Analytic sample

**Fig. 1:**
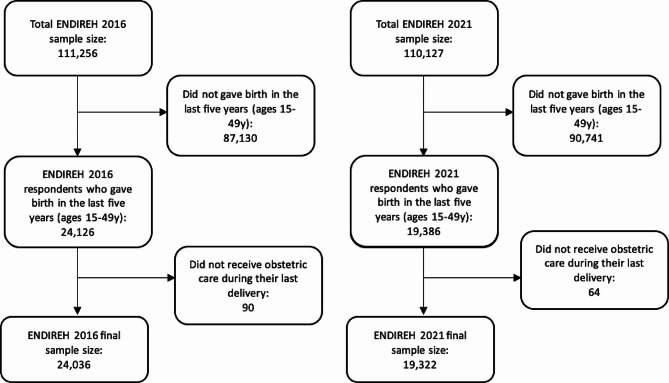
Flow charts of ENDIREH 2016 and ENDIREH 2021 final sample sizes

The probabilistic sample design for both ENDIREH 2016 and 2021 by INEGI was three-stage, stratified, and by conglomerates [41, 42]. An extended description of the sample design for both ENDIREH 2016 and 2021 has been published by INEGI [41, 42]. The total number of ENDIREH 2016 respondents was 111,256, and for ENDIREH 2021, 110,127. The study population specific for our analysis consisted of ENDIREH 2016 and 2021 respondents ages 15 to 49y who gave birth in the last five years and received obstetric care during their last childbirth. ENDIREH 2016 and 2021 respondents (ages 15-49y) who did not give birth in the last five years or who did not receive obstetric care during their last delivery were excluded from this secondary analysis. The final analytic sample size for this study was 24,036 respondents from ENDIREH 2016 and 19,322 from ENDIREH 2021 (Fig. 1).

### Variables and outcomes

#### Outcome variables

Obstetric violence in the form of non-consented care and its three different variations (forced contraceptive method or sterilization, forced signed paperwork, and forced C-section) included in ENDIREH 2016 and 2021 were the outcomes for this secondary analysis. The following questions and variables are presented as they were worded by INEGI in their English version of the 2021 ENDIREH survey [43]. While there is not an official English version of ENDIREH 2016, the questions in Spanish for both 2016 and 2021 are identical [44, 45]. A respondent was considered to have experienced non-consented care obstetric violence during their last delivery if a “yes” was provided to a question on contraceptive method or sterilization (“Were you given a contraceptive method or had an operation or sterilization to prevent you from having further children (tubal ligation-BOT) without asking or them telling you” or “Were you pressured into agreeing to have them put a device or have surgery to stop having further children?”) or on signed paperwork (“Did they force or threaten you to sign any paper without informing you what it was it or what it was for?”) or a “no” to questions related to a consented C-section (“Were you informed in a way you could understand why was it necessary to have the cesarean section?” or “Did you give permission or authorization for the cesarean section?”). The last two questions were only specific to respondents who gave birth through a C-section during their last pregnancy. Non-consented care was examined both by combining the three variations of this form of obstetric violence and by examining each of the variations separately.

#### Independent variables

##### Sociodemographic variables

For sociodemographic characteristics, these included age at the time of the survey response (< 18y, 18-25y, 26-35y, 36-45y, 46y-older), living setting (rural vs. urban), geographical region of residence, highest educational attainment (< 6th grade, middle school, high school or technical school, teacher training, college degree, or higher), ethnic self-identification (considered themselves to be Indigenous according to their culture-yes, yes-partially, no, don’t know), marital status at the time of the survey (married, cohabiting, single, separated or divorced, widow), and employment status at the time of the survey (yes, no), and type of employment (paid employee, self-employed, employer, worked without pay). As results were stratified by geographical region, the state of residence of ENDIREH 2016 and 2021 respondents was collapsed into one of the nine regions (North Pacific, Border, Central-Pacific, North Central, Central, Mexico City, State of Mexico (Estado de México), South Pacific, and Peninsula) used for the analysis. The regional stratification used for this secondary analysis followed the one used by Mexico’s National Institute of Public Health (INSP, its Spanish acronym), which groups states by their geographical proximity and population density and has been used to analyze data from other national surveys, such as the National Survey on Health and Nutrition [46]. 

##### Pregnancy and childbirth variables

For pregnancy and childbirth, the variables were number of pregnancies in the last five years; number of pregnancies in the last five years; parity in the last five years; number of stillbirths in the past five years; number of miscarriages in the past five years; type of delivery during the last childbirth (vaginal, cesarean section); health facility where prenatal care services were provided for the last pregnancy (Community health center, Mexican Institute of Social Security (IMSS) facility, Institute for Social Security and Services for State Workers (ISSSTE facility), public clinic or hospital, medical clinic or dispensary, private clinic, hospital, or medical office, other, no prenatal care received, no response); health facility where the last delivery occurred (Community health center, IMSS facility, ISSSTE facility, other state public clinic or hospital, private medical office, clinic, or hospital, other); and the type of health insurance the ENDIREH respondent had at the time of the survey (Social Security Popular/Health Institute for well-being (INSABI) Insurance, Social Security IMSS or IMSS Prospera/Bienestar, Social Security ISSSTE, private insurance, other public state institution, more than one type of insurance, does not have medical insurance, no information provided).

### Statistical analyses

Analysis proceeded in two steps:

1. Prevalence estimates were calculated using weights for each of nine geographical regions. Prevalence estimates were stratified by geographical region. Data were weighted to adjust for differences between the sample and the Mexican female population, age 15-49y, by geographical region, as certain regions were either underrepresented or overrepresented in the obstetric violence section of ENDIREH for both 2016 and 2021. For ENDIREH 2016 data, INEGI’s 2010 census information on the female population ages 15-49y of each region [47] was divided by the percentage of the ENDIREH sample for that specific region. The same was repeated with ENDIREH 2021 data using INEGI’s census data from 2020 [47]. Socioeconomic-, pregnancy-, and childbirth-specific variables of ENDIREH 2016 and 2021 respondents who experienced non-consented care during their last childbirth were reported descriptively as frequencies.
2. The next set of analyses included bivariate (crude odds ratio [COR]) and multivariable (adjusted odds ratio [AOR]) logistic regression to determine the association of sociodemographic characteristics and pregnancy and childbirth-related factors with obstetric violence in the form of non-consented care among ENDIREH 2016 and 2021 respondents. We first conducted a multicollinearity test to confirm that no multicollinearity existed among the independent variables. Then, we constructed models to estimate crude odds ratios and adjusted odds ratios with 95% confidence intervals for the association between socioeconomic and pregnancy- and childbirth-specific independent variables and non-consented care. Following the methodology of another study [16] on associated factors to obstetric violence and to avoid overfitting the model, only the variables with a *p*-value < 0.05, the value considered statistically significant, on their bivariate logistic regression were included in the multivariable logistic regression model. The data were analyzed using SPSS Version 29.0 software.

## Results

### Prevalence of non-consented care

**Fig. 2:**
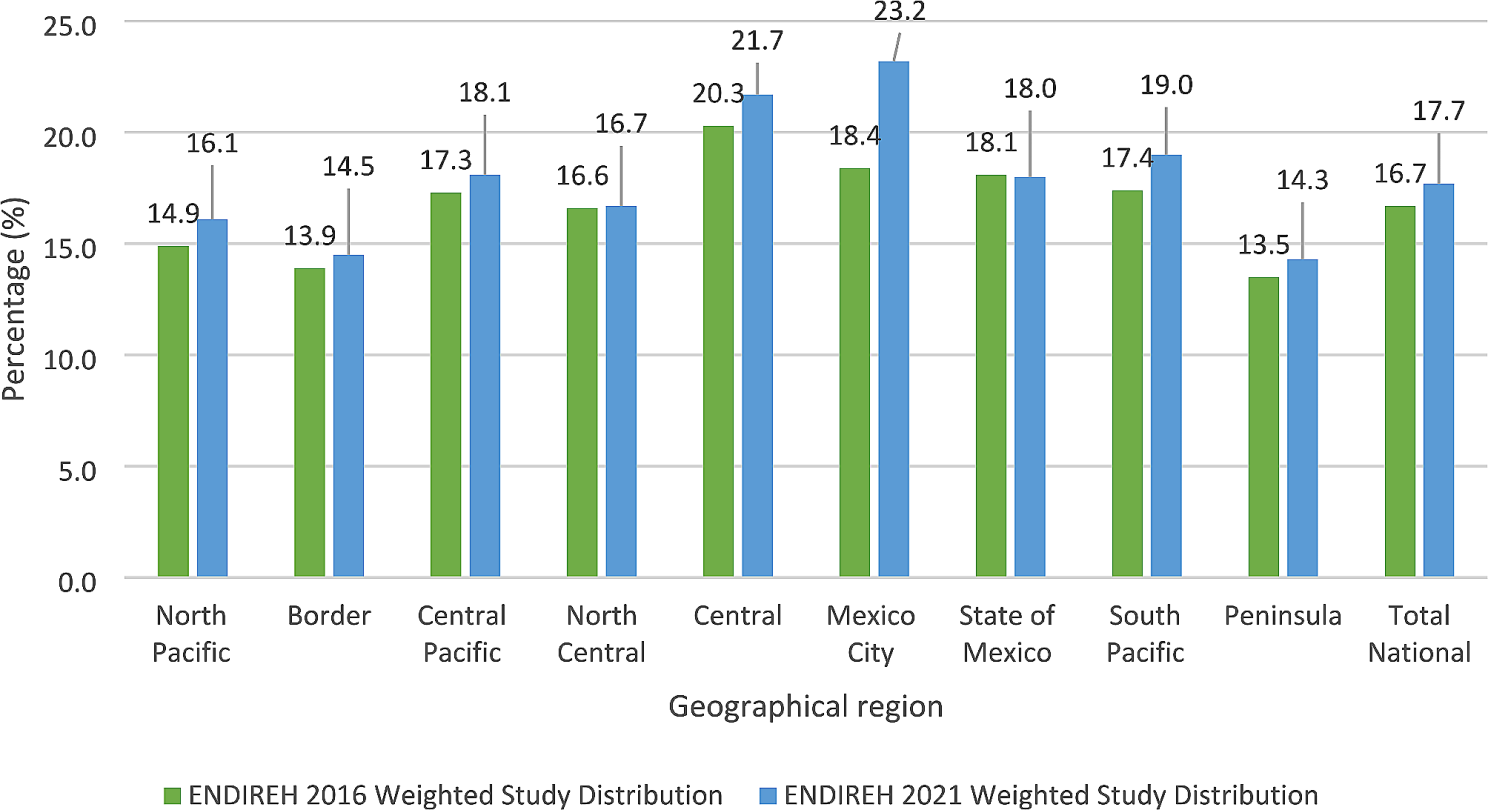
Weighted prevalence (%) of non-consented care during childbirth among women who responded to National Survey on the Dynamics of Household Relationships (ENDIREH) 2016 and 2021 stratified by geographical region (ENDIREH 2016 *n* = 24,036, ENDIREH 2021 *n* = 19,322)

A total of 3,877 and 3,823 respondents from ENDIREH 2016 and 2021, respectively, gave birth in the past five years and received obstetric care during delivery experienced non-consented care during their last childbirth. Figure 2 shows the weighted distributions of non-consented care among ENDIREH 2016 and 2021 respondents. The prevalence of non-consented care increased from 2016 to 2021 across seven of the nine geographic regions in Mexico as well as at the national level. The Central region (20.3% for ENDIREH 2016, 21.7% for ENDIREH 2021) and Mexico City (18.4% for ENDIREH 2016, 23.2% for ENDIREH 2021) had the highest prevalence of non-consented care during childbirth for both survey years. The Border (13.9% for ENDIREH 2016, 14.5% for ENDIREH 2021) and Peninsula (13.5% for ENDIREH 2016, 14.3% for ENDIREH 2021) regions had the lowest prevalence for 2016 and 2021. The rest of the results and tables will also be presented with weighted data.

### Sociodemographic characteristics and pregnancy and childbirth factors

Among respondents experiencing non-consented care, close to half of the ENDIREH 2016 and 2021 were between the ages of 26 and 35 at the time of the survey (45.2% for 2016; 49.6% for 2021). Related to marital status and educational attainment, more respondents who experienced this type of obstetric violence were married or cohabitated with a partner at the time of the survey and had completed middle school, high school, or technical school. The majority of respondents lived in urban settings (77.8% for 2016; 76.1% for 2021). For both ENDIREH 2016 and 2021 respondents, 27% were Indigenous or considered themselves partially Indigenous. While 39.2% of ENDIREH 2016 respondents who experienced non-consented care during childbirth were employed at the time of the survey, the number increased to 45.7% for ENDIREH 2021. See Table 1 for the results (at the end of the document).

**Table 1:** Weighted characteristics of National Survey on the Dynamics of Household Relationships (ENDIREH) 2016 and 2021 respondents who experienced non-consented care during last childbirth (ENDIREH 2016 *n* = 3,877, ENDIREH 2021 *n* = 3,823)

	ENDIREH 2016 respondents who experienced non-consented care during their last childbirth			ENDIREH 2021 respondents who experienced non-consented care during their last childbirth		
	% (95% CI)			% (95% CI)		
	*n* = 3877			*n* = 3823		
**Sociodemographic**						
**Age, y**						
< 18	1.9 (1.5–2.3)			1.3 (0.9–1.7)		
18–25	37.4 (35.9–38.9)			32.7 (31.1–34.3)		
26–35	45.2 (43.6–46.8)			49.6 (47.9–51.3)		
36–45	14.9 (13.8–16.0)			15.7 (14.5–16.9)		
46-older	0.5 (4.3–5.7)			0.7 (0.4-1.0)		
**Type of setting where respondent lives**						
Urban	77.8 (76.5–79.1)			76.1 (74.6–77.6)		
Rural	22.2 (20.9–23.5)			23.9 (22.4–25.4)		
**Geographical region where respondent lives**						
North Pacific	9.0 (8.1–9.9)			9.3 (8.3–10.3)		
Border	12.3 (11.3–13.3)			12.7 (11.6–13.8)		
Central Pacific	10.9 (9.9–11.9)			10.8 (9.7–11.9)		
North Central	12.7 (11.7–13.7)			13.0 (11.8–14.2)		
Central	10.2 (9.2–11.2)			9.8 (8.8–10.8)		
Mexico City	8.1 (7.2-9.0)			7.4 (6.5–8.3)		
State of Mexico (Estado de México)	14.0 (12.9–15.1)			13.9 (12.7–15.1)		
South Pacific	12.9 (11.8–14.0)			12.7 (11.6–13.8)		
Peninsula	9.9 (9.0-10.8)			10.4 (9.4–11.4)		
**Highest education attainment**						
< 6th grade	14.6 (13.5–15.7)			10.9 (9.8–12.0)		
Middle school	38.7 (37.2–40.2)			34.1 (32.5–35.7)		
High school or technical school	31.9 (30.4–33.4)			33.1 (31.5–34.7)		
Teacher training, college degree or higher	14.8 (13.7–15.9)			22.0 (20.6–23.4)		
**Literacy-knows how to read and write a note** ^a^						
Yes	88.3 (85.7–90.9)			87.6 (84.2–91.0)		
No	11.7 (9.1–14.3)			12.4 (9.0-15.8)		
**Ethnic Self-Identification-Considers herself to be Indigenous**						
Yes/Yes, partially	27.4 (26.0-28.8)			27.5 (26.0–29.0)		
No/Don’t know	72.6 (71.2–74.0)			72.5 (71.0–74.0)		
**Speaks an Indigenous language**						
Yes	6.2 (4.8–7.6)			6.9 (5.2–8.6)		
No	93.8 (92.4–95.2)			93.1 (91.4–94.8)		
**Marital Status at the time of the survey**						
Married	44.9 (43.3–46.5)			36.9 (35.2–38.6)		
Cohabiting	39.9 (38.4–41.4)			45.7 (44.0-47.4)		
Single	7.5 (6.7–8.3)			7.6 (6.7–8.5)		
Separated or divorced	7.1 (6.3–7.9)			9.1 (8.1–10.1)		
Widow	0.6 (0.4–0.8)			0.7 (0.4-1.0)		
**Employed at the time of the survey**						
Yes	39.2 (37.7–40.7)			45.7 (44.0-47.4)		
No	60.8 (59.3–62.3)			54.3 (52.6–56.0)		
**Type of employment** ^b^						
Paid employee	74.6 (72.4–76.8)			64.6 (62.2–67.0)		
Self-employed	22.9 (20.8–25.0)			31.5 (29.1–33.9)		
Employer	0.8 (0.4–1.2)			1.7 (1.0-2.4)		
Worked without pay	1.6 (1.0-2.2)			2.2 (1.5–2.9)		
**Pregnancy and childbirth**						
	Mean	SD	Range	Mean	SD	Range
**Pregnancies in the last five years** ^**c**^	1.31	0.572	1–7	1.28	0.541	1–5
**Parity in the last five years** ^**c**^	2.29	0.616	1–11	1.22	0.533	0–5
**Stillbirths in the last five years** ^**c**^	1.05	0.256	1–7	0.03	0.208	0–3
**Miscarriages in the last five years** ^**c**^	1.13	0.417	1–8	0.12	0.370	0–3
	% (95% CI)					
**Type of delivery in the last childbirth**						
Vaginal	40.4 (38.9–41.9)			40.1 (38.4–41.8)		
Cesarean section	59.6 (58.1–61.1)			59.9 (58.2–61.6)		
**Health facility where prenatal care provided in the last pregnancy**						
Community health center	34.2 (32.7–35.7)			26.0 (24.6–27.4)		
Mexican Institute of Social Security (IMSS) facility	29.4 (28.0-30.8)			30.6 (29.1–32.1)		
Institute for Social Security and Services for State Workers (ISSSTE facility)	2.3 (1.8–2.8)			2.2 (1.7–2.7)		
Public clinic or hospital	15.8 (14.7–16.9)			15.9 (14.7–17.1)		
Medical clinic or dispensary	1.4 (1.0-1.8)			0.9 (0.6–1.2)		
Private clinic, hospital, or medical office	10.2 (9.2–11.2)			15.2 (14.1–16.3)		
Other^d^	6.0 (5.3–6.7)			8.8 (7.9–9.7)		
No prenatal care received	0.6 (0.4–0.8)			0.4 (0.2–0.6)		
No response	0.1 (0.0-0.2)			0		
**Health facility where last delivery occurred**						
Community Health Center	10.3 (9.3–11.3)			8.2 (7.3–9.1)		
IMSS facility	35.6 (34.1–37.1)			36.6 (35.0-38.2)		
ISSSTE facility	2.7 (2.2–3.2)			2.6 (2.1–3.1)		
Other state public clinic or hospital	39.5 (38.0–41.0)			37.5 (35.8–39.2)		
Private medical office, clinic, or hospital	10.3 (9.3–11.3)			13.7 (12.5–14.9)		
Other^e^	1.6 (1.2-2.0)			1.4 (1.0-1.8)		
**Current medical service affiliation**						
Popular Social Security/Health Institute for well-being (INSABI) Insurance^f^	47.9 (46.3–49.5)			17.4 (16.2—18.6)		
Social Security IMSS or IMSS Prospera/Bienestar^g^	29.8 (28.4–31.2)			37.4 (35.9–38.9)		
Social Security ISSSTE	3.0 (2.5–3.5)			3.5 (2.9–4.1)		
Private Insurance	0.5 (0.3–0.7)			0.4 (0.2–0.6)		
Other public state institution^h^	0.6 (0.4–0.8)			2.2 (1.7–2.7)		
More than one	3.7 (3.1–4.3)			1.1 (0.8–1.4)		
Does not have medical insurance	9.0 (8.1–9.9)			38.0 (36.5–39.5)		
No information provided	5.5 (4.8–6.2)			0		

^a^For respondents who completed 6th grade or less

^b^For respondents employed at the time of the survey

^c^In the last five years for ENDIREH 2016 is between 2011 and 2016 and for ENDIREH 2021 is between 2016 and 2021

^d^Includes midwives, healers, physicians at pharmacies, and receiving more than one type of service

^e^Includes at home with a midwife or healer

^f^ Social Security Popular (Seguro Popular, in Spanish) evolved into the Health Institute for well-being (Instituto de Salud para el Bienestar, INSABI, in Spanish) in 2020 [62].

^g^ IMSS Prospera evolved into IMSS Bienestar in 2019 [63].

^h^Includes insurance from PEMEX, Marines, and Defense

Regarding the pregnancy and childbirth factors of respondents who experienced non-consented care during their last childbirth, the average number of pregnancies in the last five years was relatively the same between 2016 and 2021 (1.31 for 2016, 1.28 for 2021). C-section was the delivery method for 59% of the deliveries for ENDIREH 2016 and 2021. Community health centers and Mexican Institute of Social Security (IMSS) facilities, which provide healthcare services to formally employed members of the private sector with insurance for these facilities, and other public clinics or hospitals were the most common locations where ENDIREH 2016 and 2021 respondents received their prenatal care and their place of delivery for their last pregnancy. While the type of current medical service affiliation was different among ENDIREH 2016 and 2021 respondents, more than half of ENDIREH 2016 respondents who experienced non-consented care had Social Security IMSS (insurance to receive services at IMSS facilities) or IMSS Prospera insurance or Popular Social Security (types of insurance for marginalized populations who do not have access to other forms of insurance) at the time of the survey (77.7%), and 75.4% of ENDIREH 2021 respondents who experienced non-consented care during their last childbirth had Social Security IMSS or IMSS Bienestar (formerly IMSS Prospera) or did not have any form of medical insurance at the time of the interview. See Table 1 for the results (located at the end of the document).

### Prevalence of specific forms of non-consented care

Table 2 reports findings on the prevalence of specific forms of non-consented care. The prevalence of forced contraception or sterilization without knowledge or authorization was higher in 2016, compared to 2021, for the North Pacific (26.9% vs. 25.4%), North Central (20.4% vs. 16.2%), Central (23.2% vs. 22.7%), Mexico City (23.8% vs. 22.6%), State of Mexico (Estado de México) (33.0% vs. 23.1%), and Peninsula (22.9% vs. 21.6%). Pressure to get a contraceptive method or sterilization was higher among ENDIREH 2021 respondents compared to ENDIREH 2016 respondents in six out of the nine geographical regions. Forcing or threatening to sign paperwork was the form of non-consented care with the lowest prevalence in both ENDIREH 2016 and 2021; this specific form of non-consented care saw a minor decrease from 2016 to 2021 in seven out of the nine regions. Among the two questions related to C-sections, the prevalence of non-consented care during the last childbirth was higher among ENDIREH 2016 respondents in most geographical regions. Not being informed about the need for a C-section was higher in the Border region in 2016 compared to 2021 (56.0% vs. 39.0%), while in the Central Pacific region, the prevalence was lower in 2016 compared to 2021 (34.2% vs. 43.5%). Related to providing authorization for C-sections, the highest prevalence differences between 2016 and 2021 were in the North Pacific (37.4% vs. 26.5%), Mexico City (40.2% vs. 26.7%), and the Central Pacific (43.0% vs. 29.0%) regions.

**Table 2:** Weighted Prevalence (% and 95% CI) of different forms of non-consent care experienced by ENDIREH 2016 and 2021 respondents stratified by geographical region (ENDIREH 2016 *n* = 3,877, ENDIREH 2021 *n* = 3,823)

	Forced contraceptive method or sterilization without knowledge or authorization (% and 95% CI)		Pressure to get a contraceptive method or sterilization (% and 95% CI)		Forced or threatened to sign paperwork (% and 95% CI)		Not informed about the need of a Cesarean section (% and 95% CI)^a^		Did not provide authorization for Cesarean section (% and 95% CI)	
	ENDIREH 2016	ENDIREH 2021	ENDIREH 2016	ENDIREH 2021	ENDIREH 2016	ENDIREH 2021	ENDIREH 2016	ENDIREH 2021	ENDIREH 2016	ENDIREH 2021
North Pacific	26.9 (25.5–28.3)	25.4 (24.0-26.8)	48.3 (46.7–49.9)	56.6 (55.0-58.2)	6.0 (5.3–6.7)	7.9 (7.0-8.8)	42.3 (40.7–43.9)	40.0 (38.4–41.6)	37.4 (35.9–38.9)	26.5 (25.1–27.9)
Border	23.1 (21.8–24.4)	27.3 (25.9–28.7)	50.9 (49.3–52.5)	47.2 (45.6–48.8)	8.8 (7.9–9.7)	7.9 (7.0-8.8)	56.0 (54.4–57.6)	39.0 (37.5–40.5)	36.7 (35.2–38.2)	38.1 (36.6–39.6)
Central Pacific	15.9 (14.7–17.1)	23.4 (22.1–24.7)	57.7 (56.1–59.3)	58.9 (57.3–60.5)	10.2 (9.2–11.2)	7.6 (6.8–8.4)	34.2 (32.7–35.7)	43.5 (41.9–45.1)	43.0 (41.4–44.6)	29.0 (27.6–30.4)
North Central	20.4 (19.1–21.7)	16.2 (15.0-17.4)	60.1 (58.6–61.6)	66.4 (64.9–67.9)	9.8 (8.9–10.7)	8.2 (7.3–9.1)	43.7 (42.1–45.3)	39.6 (38.0-41.2)	35.5 (34.0–37.0)	33.3 (31.8–34.8)
Central	23.2 (21.9–24.5)	22.7 (21.4–24.0)	61.9 (60.4–63.4)	59.0 (57.4–60.6)	7.8 (7.0-8.6)	6.2 (5.4-7.0)	37.7 (36.2–39.2)	29.1 (27.7–30.5)	40.4 (38.9–41.9)	38.7 (37.2–40.2)
Mexico City	23.8 (22.5–25.1)	22.6 (21.3–23.9)	62.5 (61.0–64.0)	58.0 (56.4–59.6)	13.7 (12.6–14.8)	12.8 (11.7–13.9)	41.9 (40.3–43.5)	31.7 (30.2–33.2)	40.2 (38.7–41.7)	26.7 (25.3–28.1)
State of Mexico (Estado de México)	33.0 (31.5–34.5)	23.1 (21.8–24.4)	52.0 (50.4–53.6)	56.8 (55.2–58.4)	12.4 (11.4–13.4)	14.7 (13.6–15.8)	47.5 (45.9–49.1)	41.3 (39.7–42.9)	39.7 (38.2–41.2)	30.3 (28.8–31.8)
South Pacific	25.8 (24.4–27.2)	26.6 (25.2–28.0)	56.0 (54.4–57.6)	59.1 (57.5–60.7)	10.2 (9.2–11.2)	5.3 (4.6-6.0)	41.4 (39.8–43.0)	27.7 (26.3–29.1)	35.8 (34.3–37.3)	38.0 (36.5–39.5)
Peninsula	22.9 (21.6–24.2)	21.6 (20.3–22.9)	39.5 (38.0–41.0)	43.1 (41.5–44.7)	8.1 (7.2-9.0)	6.4 (5.6–7.2)	42.0 (40.4–43.6)	41.9 (40.3–43.5)	50.7 (49.1–52.3)	45.9 (44.3–47.5)
Total National	24.1 (22.8–25.4)	23.2 (21.9–24.5)	53.4 (51.8–55.0)	56.2 (54.6–57.8)	9.7 (8.8–10.6)	8.6 (7.7–9.5)	43.2 (41.6–44.8)	37.3 (35.8–38.8)	39.9 (38.4–41.4)	34.5 (33.0–36.0)

### Factors associated with non-consented care

Unadjusted and adjusted associations of sociodemographic characteristics and pregnancy and childbirth factors with experiencing non-consented care are shown in Table 3 (located at the end of the document). Ethnic self-identification, number of pregnancies in the last five years, parity in the last five years, and miscarriages in the last five years were not included in the adjusted model based on the *p*-values of their bivariate logistic regressions. In adjusted models, compared to those ≤18 years old, ENDIREH 2016 and 2021 respondents 26-35y and 36-45y had significantly higher odds of non-consented care during their last childbirth. This was also true for ENDIREH 2021 respondents 46 years of age and older. Additionally, ENDIREH 2016 and 2021 respondents living in rural (vs. urban) settings, having one or more stillbirths (vs. none), and having a vaginal delivery (vs. C-section) also had significantly higher odds of non-consented care during their last childbirth. ENDIREH 2016 and 2021 respondents who gave birth at a private clinic, hospital, or medical office (vs. community health center) or had a medical service affiliation at the time of the survey at other public state institutions (vs. Social Security IMSS) had significantly higher odds of experiencing non-consented care during their last childbirth. ENDIREH 2016 respondents having private insurance, Social Security ISSSTE (a type of insurance for formally employed employees of the public sector), or Prospera at the time of the survey also had significantly higher odds of non-consented care during their last childbirth (vs. Social Security IMSS).

**Table 3:** Association between sociodemographic characteristics, pregnancy, and childbirth factors and experiencing non-consented care during childbirth among ENDIREH 2016 and 2021 respondents

		COR (95% CI)	AOR (95% CI)
**Age, y**			
	< 18	1	1
	18–25	1.06 (0.87–1.30)	1.13 (0.92–1.40)
	26–35	1.33 (1.09–1.63)**	1.47 (1.19–1.82)***
	36–45	1.44 (1.17–1.77)***	1.63 (1.31–2.02)***
	46-older	1.44 (0.98–2.11)	1.60 (1.08–2.37)*
**Setting**			
	Urban	1	1
	Rural	1.19 (1.12–1.26)***	1.16 (1.09–1.24)***
**Geographical region**			
	North Pacific	1	1
	Border	1.11 (0.99-0.1.24)	1.06 (0.94-0.1.19)
	Central Pacific	0.85 (0.76–0.95)**	0.81 (0.72–0.91)***
	North Central	0.92 (0.82–1.02)	0.84 (0.75–0.94)**
	Central	0.69 (0.62–0.77)***	0.64 (0.57–0.72)***
	Mexico City	0.71 (0.63–0.80)***	0.65 (0.57–0.73)***
	State of Mexico (Estado de México)	0.83 (0.77–0.92)***	0.72 (0.64–0.80)***
	South Pacific	0.83 (0.74–0.92)***	0.72 (0.64–0.81)***
	Peninsula	1.14 (1.00-1.28)*	1.07 (0.94–1.21)
**Educational attainment**			
	Up to 6th grade	1	
	Middle school	0.89 (0.83–0.97)**	1.00 (0.92–1.09)
	High school or technical school	0.78 (0.72–0.85)***	0.89 (0.81–0.97)**
	Teacher training, college degree or higher	0.89 (0.81–0.97)*	0.82 (0.74–0.91)***
**Ethnic-Self Identification-Considers herself to be Indigenous**			
	Yes/Yes, partially	1	
	No/Don’t know	1.00 (0.95–1.06)	
**Employed at the time of the survey**			
	Yes	1	1
	No	1.07 (1.01–1.12)*	1.03 (0.97–1.09)
**Marital Status**			
	Cohabitating	1	1
	Separated or divorced	0.94 (0.85–1.04)	0.94 (0.85–1.04)
	Widow	0.91 (0.66–1.25)	0.80 (0.58–1.12)
	Married	1.13 (1.07–1.19)***	1.02 (0.96–1.08)
	Single	0.84 (0.76–0.93)***	0.89 (0.80–0.99)*
**Number of pregnancies in the last 5 years**			
	1	1	
	> 1	1.00 (0.95–1.06)	
**Parity in the last five years**			
	0	1	
	1	0.96 (0.70–1.32)	
	> 1	1.03 (0.75-0.1.41)	
**Stillbirths in the last five years**			
	0	1	1
	≥1	1.07 (1.01–1.12)*	1.11 (1.05–1.18)***
**Miscarriages in the last five years**			
	0	1	
	≥1	1.03 (0.97–1.08)	
**Type of delivery in the last childbirth**			
	Cesarean section	1	1
	Vaginal	2.00 (1.91–2.11)***	2.42 (2.29–2.55)***
**Location of prenatal care provider**			
	Community health center	1	1
	IMSS facility	0.81 (0.76–0.87)***	1.15 (1.03–1.28)*
	ISSSTE facility	1.10 (0.93–1.30)	0.93 (0.72–1.21)
	Public clinic or hospital	0.99 (0.91–1.07)	1.04 (0.96–1.13)
	Medical clinic or dispensary	1.04 (0.82–1.31)	0.96 (0.76–1.22)
	Private clinic, hospital, or medical office	1.58 (1.46–1.71)***	1.05 (0.94–1.17)
	Other^a^	0.97 (0.87–1.07)	0.81 (0.72–0.91)***
	No prenatal care received	1.26 (0.91–1.74)	1.08 (0.77–1.52)
**Place of childbirth delivery**			
	Community Health Center	1	1
	IMSS facility	0.69 (0.63–0.76)***	0.64 (0.57–0.73)***
	ISSSTE facility	1.05 (0.88–1.25)	1.20 (0.94–1.55)
	Other state public clinic or hospital	0.86 (0.78–0.94)**	0.88 (0.80–0.97)*
	Private medical office, clinic, or hospital	1.93 (1.74–2.15)***	2.69 (2.36–3.06)***
	Other^b^	2.54 (2.06–3.14)***	2.16 (1.73–2.70)***
**Current medical service affiliation**			
	Social Security IMSS	1	1
	Social Security ISSSTE or Prospera/Bienestar^c^	1.43 (1.24–1.64)***	1.15 (0.95–1.39)
	Social Security Popular/Health Institute for well-being (INSABI) insurance^d^	1.11 (1.05–1.18)***	0.93 (0.86–1.02)
	Other public state institution^e^	1.67 (1.34–2.09)***	1.33 (1.06–1.68)*
	Private Insurance	2.18 (1.55–3.06)***	1.15 (0.81–1.64)
	More than one	1.24 (1.05–1.46)**	0.99 (0.83–1.17)
	Does not have medical insurance	1.16 (1.08–1.24)***	0.97 (0.90–1.05)

^a^Includes midwives, healers, physicians at pharmacies, and receiving more than one type of service

^b^Includes at home with a midwife or healer

^c^IMSS Prospera evolved into IMSS Bienestar in 2019 [63].

^d^Social Security Popular (Seguro Popular, in Spanish) evolved into the Health Institute for well-being (Instituto de Salud para el Bienestar, INSABI, in Spanish) in 2020 [62].

^e^Includes insurance from PEMEX, Marines, and Defense

* *p* < 0.05 ** *p* < 0.01 *** *p* < 0.001

COR: Crude Odds Ratio; AOR: Adjusted Odds Ratio; CI: Confidence Interval

Several factors were associated with lower odds of experiencing non-consented care. Compared to the North Pacific region, respondents living in the Central Pacific, Central, Mexico City, State of Mexico (Estado de México), and South Pacific regions had significantly lower odds of experiencing non-consented care. Respondents whose marital status was single (vs. cohabitating) at the time of the survey or whose educational attainment was high school or technical school and teaching training, college degree, or higher (vs. 6th grade or less) had lower odds of experiencing non-consented care. Receiving prenatal care for the last pregnancy at other types of facilities or an IMSS facility (vs. community health center) also had lower odds of experiencing non-consented care during the last childbirth for ENDIREH 2021 respondents.

## Discussion

This study aimed to investigate the prevalence of a specific form of obstetric violence, non-consented care, among women in Mexico and to assess the relationship between sociodemographic characteristics, pregnancy and childbirth factors, and the odds of reporting non-consented care. This study builds on previous research on obstetric violence in Mexico using ENDIREH data [13], by comparing for the first time ENDIREH results from 2016 to 2021 and stratifying by geographic regions of the country. We found the overall prevalence of non-consented care during childbirth increased from 2016 to 2021 at the national level and in seven out of nine geographical regions. Related to specific forms of non-consented care during childbirth, we also documented that forcing or threatening a woman to sign paperwork was the least common form for both years, while pressuring them to get a contraceptive method or sterilization during the childbirth process, not informing them about the need for a C-section, and not allowing women to provide authorization for this specific process were the most common forms of non-consented care.

Our findings show that the Central region had the second highest prevalence of experienced non-consented care among ENDIREH 2016 recipients and the highest for ENDIREH 2021. These findings add to a previous observational study on respect and evidence-based birth care in different states in Mexico, which found that in one of the states in the Central region, Hidalgo, the instruments used for hospitals in this state did not collect information about informed consent and dignified care [29]. The higher prevalence of non-consented care in the Central and South Pacific regions also coincided with some of the states in the country with the highest marginalization indices. Two (Veracruz and Hidalgo) of the three Mexican states that make up the Central region have high levels of marginalization, while three (Chiapas, Guerrero, and Oaxaca) out of the four states that make up the region of the South Pacific are considered to have very high levels of marginalization [48]. The marginalization index measures how the lack of access to education, inadequate housing, and lack of assets impact a specific population [49]. Previous research also found women from highly marginalized states suffer high levels of physical, psychological, sexual, and economic violence during pregnancy [50]. As ENDIREH collects information not just on obstetric violence but other types of gender-based violence, future studies should examine the relationship between non-consented care and other forms of obstetric violence with physical, psychological, sexual, and economic violence at the interpersonal level nationally and at the regional or state level.

The prevalence of the different sociodemographic, pregnancy, and childbirth characteristics analyzed in this study was similar among ENDIREH 2016 and 2021 respondents, except for the current medical service affiliation. This is due to the changes in the health care system in the last few years, as Social Security Popular (Seguro Popular, in Spanish) evolved into the Health Institute for well-being (Instituto de Salud para el Bienestar, INSABI, in Spanish) in 2020, reducing the levels of health coverage among the Mexican population, as seen in our results. Previous research on ENDIREH 2016 found that obstetric violence and non-consented care were more common among women who lived in urban regions, were single, younger, did not speak an Indigenous language, had higher educational attainment, and gave birth during their last childbirth at state public hospitals, a Social Security Institute, or community public health centers [13]. We found similar results not just for 2016, but also for 2021; as they show for both ENDIREH 2016 and 2021, non-consented care was more prevalent among women who live in urban regions, those who do not identify as Indigenous, those who considered themselves Indigenous and did not speak an Indigenous language, and those whose last delivery occurred at an IMSS facility or another state public clinic or hospital. However, for both 2016 and 2021, our results showed that the age of the women with a higher prevalence of non-consented care extended from 18 to 35 (at the time of the survey). While, regarding educational attainment, our results showed that the women with the highest prevalence of non-consented care were had either completed middle school, high school, or technical school. The potential reasons for these differences between ENDIREH 2016 results from our research and previous published literature are the way these sociodemographic, pregnancy, and childbirth factors were stratified, the sample sizes used to evaluate the prevalence of non-consented care, and the fact that our results are shown as weighted estimates adjusted by geographical region. Regarding the living environment, previous literature has found obstetric violence to be common among rural communities in Mexico [29, 51]; however, our results found a higher prevalence among urban populations. A study done in Ecuador also found a higher prevalence of obstetric violence among women living in urban areas [52]. Further research is required to better understand how different social inequities lead to obstetric violence against women in Mexico or to address the possibility of women from rural or Indigenous populations underreporting this type of violence in ENDIREH.

Related to the association between non-consented care during childbirth and sociodemographic characteristics and pregnancy and childbirth factors, our findings suggest the place of delivery as a factor highly associated with this type of obstetric violence among ENDIREH 2016 and 2021 respondents. Results from ENDIREH are the first to provide an analysis at the national level of this type of association, as previous research in Mexico has only been completed qualitatively or at the local level in one or a few numbers of hospitals [7, 11, 19, 29] without the possibility of comparing the different and unique types of health care settings in this country. The prevalence of C-sections in Mexico is high, the second highest in the Americas [53]. Results from our study confirm this, as close to 60% of ENDIREH 2016 and 2021 respondents experienced non-consented care delivered via C-section. According to literature, different Mexican institutions, such as IMSS and private facilities, have a C-section prevalence higher than what is recommended by the WHO [54]. Previous data has shown that women and physicians in Mexico prefer C-section as the delivery method due to its convenience and being considered safer than a vaginal delivery [55, 56]. Still, our results also show that ENDIREH respondents who had a vaginal delivery had greater odds of experiencing non-consented care than those who delivered via C-section; however, those who received prenatal care services or gave birth at IMSS or private facilities had higher odds of experiencing non-consented care. Future ENDIREH surveys could further examine the reasons behind decisions made by women related to pregnancy and childbirth factors, such as prenatal care services received and type of delivery.

Our findings are consistent with research that shows how obstetric violence is the result of a continuum of visible and invisible factors at the different levels of society [28]. These factors include the degree of autonomy and empowerment of the women on different childbirth choices, such as the type of delivery (vaginal or C-section), prenatal care received, and delivery location, as well as the contributions from the medical providers and major social institutions such as the health care facilities and the governments in charge of enforcing and implementing laws. Regarding laws implemented and enforced, our study found that overall prevalence of non-consented care decreased in one of the nine regions from 2016 to 2021, while forcing or threatening to sign paperwork, not informing about the need for a C-section, and not providing authorization for C-section decreased among most regions from 2016 to 2021, while pressure to get a contraceptive method or sterilization increased at the national and regional level. The partial decrease of different forms of non-consented care from 2016 to 2021 may be attributed to the changes in Mexican law at the national and state levels related to the care of women during pregnancy, childbirth, the postpartum period, and the newborn between those years. However, the increase in some forms of non-consented care raises concerns about the implementation of the new law. A recent study completed in Mexico City found that health personnel are aware of and understand the new laws towards eliminating obstetric violence; however, they continue to witness or perform activities that constitute obstetric violence [57]. Reasons in the literature that have been found behind obstetric violence at health care facilities in Mexico include institutional barriers such as a shortage of specialists and the additional training required, as well as the under-resourced and strained health systems in Mexico, such as a lack of space and infrastructure [58]. Formal and constant supervision at every health center to prevent obstetric violence, as well as accountability mechanisms, are needed to reassure that these laws are followed.

Mexico joins at least two countries in Latin America, Venezuela and Argentina, that have laws against obstetric violence [28]. However, in these two countries, the laws are aimed more at identifying and reporting this type of violence against women than preventing it, and little is known about their effectiveness in reducing this type of violence against women [59]. Mexico has an opportunity to take the lead in Latin America on developing and enforcing a definite legal framework at the national and state levels that defines obstetric violence and laws that protect women during pregnancy and childbirth through the respectful care of medical professionals and institutions to prevent this type of violence against women from occurring. Our results suggest that to reduce this problem, there is a need to strengthen health systems for all types of public and private health facilities, paying special attention to the geographical regions and populations that have experienced higher reported cases of this structural problem.

This secondary analysis has several strengths worth mentioning. First, we had a large sample size which increases the statistical power. Second, both ENDIREH 2016 and 2021 are representative of the state and national level. Third, weighting the results allowed us to account for underrepresented geographical regions that were sampled. And, finally, while obstetric violence and its specific form of non-consented care during childbirth are complex events to measure and there are no validated or standardized tools for this, ENDIREH 2016 and 2021 follow the same rigorous methodology, allowing us to build on the validity of this national household survey.

We acknowledge several limitations of this study, including that self-reporting of any form of sensitive information, such as violence, is prone to different types of biases, such as recall and social desirability bias [60, 61]. ENDIREH only asks about the last childbirth experience, excluding potential events of obstetric violence in previous deliveries. Recall bias from ENDIREH respondents who have given birth more than once could have potentially combined the experiences of their different deliveries when answering this survey. ENDIREH only interviews one woman per household, which potentially excludes other women who suffered from this type of violence from being part of the survey. Women who last gave birth more than five years ago (at the time of the survey) are excluded from answering questions related to obstetric violence. And, finally, using proxy information from the time of the survey for some sociodemographic characteristics (such as age, marital status, and employment status) rather than at the time of the last childbirth is a major limitation of this analysis.

## Conclusion

Results from this secondary analysis showed an increase from 2016 to 2021 in non-consented care during childbirth, and in one of its variations, pressure to get a contraceptive method or sterilization. While there is a decrease in the prevalence of forced contraceptive method or sterilization without knowledge or authorization and non-consented C-sections, more than 15% of ENDIREH respondents who gave birth in the last five years experienced at least one variation of non-consented care. More research on obstetric violence, such as expanding the obstetric violence section in the next ENDIREH, is mandatory to further understand why the prevalence of some non-consented practices has increased while others have decreased, as well as to have greater evidence on the risk factors behind this type of violence and to tailor solutions at the different systems (individuals, communities, healthcare providers, service delivery locations, state and national laws and policies) to each geographical region and each type of population affected by it.

## Data Availability

The datasets used for analysis are publicly available in the INEGI website (for ENDIREH 2016, http://en.www.inegi.org.mx/programas/endireh/2016/#Open_data; for ENDIREH 2021, http://en.www.inegi.org.mx/programas/endireh/2021/#Open_data).

## Abbreviations

AOR: Adjusted Odds Ratio
C-section: Cesarean section
CI: Confidence Interval
COR: Crude Odds Ratio
ENDIREH 2016: National Survey on the Dynamics of Household Relationships 2016
ENDIREH 2021: National Survey on the Dynamics of Household Relationships 2021
IMSS: Mexican Institute of Social Security
INEGI: National Institute of Statistics and Geography
INSP: National Institute of Public Health
ISSSTE: Institute for Social Security and Services for State Workers
WHO: World Health Organization
